# Experimental Investigation of Cutting Vibration during Micro-End-Milling of the Straight Groove

**DOI:** 10.3390/mi11050494

**Published:** 2020-05-13

**Authors:** Lijie Ma, Ian Howard, Minghua Pang, Zhankui Wang, Jianxiu Su

**Affiliations:** 1 School of Mechanical and Electrical Engineering, Henan Institute of Science and Technology, Xinxiang 453003, China; Pangminghua909@163.com (M.P.); luckywzk@126.com (Z.W.); dlutsu2004@163.com (J.S.); 2 Department of Mechanical Engineering, Curtin University, Perth 6102, Australia; i.howard@curtin.edu.au

**Keywords:** micro-end-milling, cutting vibration, time–domain waveform, frequency response, milling parameters, acceleration vibration, correlation, relationship model

## Abstract

Micro-end-milling is a cutting technology that removes redundant material from machined workpieces by small-diameter end mills, and is widely used to manufacture miniature complex parts. During micro-end-milling, the cutting vibration caused by weak tool rigidity and high spindle speed is known as a key factor for decreasing machined quality and accelerating tool failure. This study reports on experiments of micro-end-milling of the straight groove for AISI 1045 steel. The waveform characteristics of acceleration vibration were revealed, the relationship between acceleration and milling parameters were analyzed and two types of relationship models were developed. The results show that, during micro-end-milling of the straight groove, the components of acceleration vibration from largest to smallest are in turn the transverse acceleration *α_Y_*, the feed acceleration *α_X_* and the axial acceleration *α_Z_*. Compared with feed velocity *v_f_* and axial depth of cut *a_p_*, the spindle speed *n* has the highest influence on cutting vibration. The response surface model of acceleration vibration was shown to have a higher prediction accuracy compared to the power function model and is more suitable for the prediction and control of cutting vibration during micro-end-milling.

## 1. Introduction

As a result of good material adaptability and high material removal rate, micro-cutting has been shown to be an effective microsystem manufacturing technology that is particularly suitable for machining microstructure parts and miniature parts [1]. Micro-end-milling is one of the main methods of micro-cutting technology, which is very flexible for manufacturing complex three-dimensional geometries [2,3]. In order to be practical for industrial application, the goals of micro-end-milling are to achieve good machining accuracy, low surface roughness and long tool life. However, the characteristics of micro-end-milling, such as small cutting area, weak tool rigidity and high spindle speed, often result in drastic cutting vibration, which has been a major obstruction in achieving higher productivity, better accuracy and lower cost [4,5,6]. Hence, the detection, analysis and suppression of cutting vibration have become an important research field for micro-end-milling.

The machining chatter is a severe vibration phenomenon caused by the combination of cutting conditions and machine tools, and the machining dynamics is a key discipline to determine a chatter-free cutting condition. There has been some research works done on the dynamic foundations of micro-milling. Wojciechowski [7] developed a new cutting force prediction model of micro-end-milling which considers the chip thickness accumulation phenomenon resulting from burnishing and elastic recovery in the micro-cutting area. The instantaneous and average milling force can be predicted accurately by the developed model, which provides a foundation for the dynamic analysis of micro-milling. Zhang [8] proposed a cutting force model considering process nonlinearities and process damping, obtained the dynamic parameters of the micro-milling system by the receptance coupling method, and then analyzed the chatter stability of micro-end-milling. Martin [9,10] built a dynamic model of micro-end-milling considering the tool structure and process faults and investigated the effects that the process faults have on the machining chatter. His research showed that the influence of the unbalance from process faults on dynamic stability is significant with the increase of spindle speed. Tajalli [11], based on the strain gradient elasticity theory, presented a size-dependent formulation and analytic approach of studying forced vibration and chatter instability observed in micro-milling operations. Shi [12] investigated the effects of gyroscopic motion and mode interaction on the dynamics of micro-end-milling due to regenerative chatter, where the results showed that the mode interaction strongly affects the dynamics and the chatter stability.

Meanwhile, some progress has been achieved in the prediction and suppression of machining chatter. Park [13] investigated the dynamics of the tool tip during high speed micro-milling, proposed a robust stability prediction method with process damping and achieved a better prediction result. Mittal [14] studied the influence of flood lubrication on the dynamic stability during high speed micro-milling of Ti6Al4V. The result showed that flood lubrication can enhance the stability limit when the spindle speed is higher than 47,000 rpm, and that the influence of lubrication mode is more significant with the increase of spindle speed and feed per tooth. Saleh [15] considered the micro-milling system as a two-degree of freedom system and applied linear and nonlinear vibration absorbers to suppress regenerative chatter. The results indicated that the nonlinear absorber with quadratic damping behaviors is more effective to enlarge the stable zone. Ko [16,17] studied the effect of workpiece ultrasonic vibration on the milling chatter and found that the cusp error and chatter marks can be reduced significantly by the choice of suitable vibration mode and parameters. Wang [18] proposed a selection method of tool overhang length based on the stability analysis and verified the reliability of this method by the experiments of micro-milling.

Many research works showed that, even in the case of chatter-free machining, the cutting vibration also has a great effect on the process effect of micro-end-milling. The research of Moges [19] showed that the tool deflection and the vibration of the tool tip can seriously deteriorate the cutting force and surface quality of micro-end-milling. Hsieh [5] developed a vibration monitoring system for micro-milling by acquiring the vibration signals of the spindle, then studied the relationship of spindle vibration and tool wear via a backpropagation neural network. The results showed that the cutting vibration is an important factor influencing tool life, and that the frequency domain signal of cutting vibration can be used for monitoring micro-tool failure with proper feature extraction and classifier design. Weiner [20] studied the effect of tool runout on cutting vibration and surface quality by using the dynamic simulation method, with the results showing that, although the tool runout did not cause cutting chatter, it increased the vibration amplitude in the direction perpendicular to the feed motion which directly weakens the surface quality. Li [21] developed a hybrid 2D cutting force model for micro-milling which investigated the impact of tool runout and tool trajectory on the cutting force. The comparison of the predicted and measured results showed that the developed model has higher predict accuracy. Attanasio [22] studied the effect of tool runout on the cutting force, chip flow and chip shapes by the FE simulation method and proved its validity by the micro-milling experiments. Wojciechowski [23] carried out the micro-milling experiments of hardened alloy steel and studied the effect of the tool axis inclination angle and feed per tooth on the milling vibration during micro ball end milling. The result showed that these two parameters significantly affect the vibration amplitude and surface roughness, and that the ploughing action in the micro-milling area can be reduced when a larger tool axis inclination angle and smaller feed per tooth are selected, so that the machining quality can be improved. Mustapha [24] developed a hybrid analytical model for estimating the transverse vibration response of micro-end-milling and compared the response profiles from the experiment and the developed model, which showed reasonably close similarity. Lu [25] built a surface roughness prediction model using the measured results of tool vibration and predicted the floor surface roughness in the micro-milling of straight grooves with good accuracy.

Furthermore, when milling complex surfaces and free-form surfaces, the chatter stability and cutting vibration are also important issues that must be considered. Pelayo [26] provided a time domain model for inclined milling operations and applied this model to investigate the tool’s static and dynamic cutting behavior, with the result showing that the proposed model can accurately predict cutting force and machining chatter. Liu [27] proposed a milling chatter stability prediction method for milling a free-form surface based on the time–domain behavior and studied the influence of surface curvature and tool lead angle on the chatter stability domain. The results showed that the time–domain simulation method can reveal the instability mechanism during milling of a free-form mold. Toh [28], based on the experiments of high-speed finish milling for inclined hardened steel, found that the cutter path orientation directly influences the tool vibration, and further impacts the tool life and surface quality. Wojciechowski [29,30] developed a cutter’s displacement (vibration) model of ball end milling that included surface inclination angle, radial run out and tool deflection, etc., and studied comparatively the simulated and experimental results of cutter displacements. The results showed that the cutter’s vibration is significantly affected by radial run out and surface inclination angle, and that the selection of appropriate tool axis slope can achieve high machining quality.

According to the above analysis, during either micro-end-milling or end-milling of complex surfaces, vibration control is always an important issue for improving machining quality and extending tool life. The groove is the basic surface shape of forming miniature three-dimensional parts. The objective of this study is to disclose the vibration characteristics during micro-end-milling of the straight groove and find a method that is suitable for the vibration prediction and control of micro-end-milling. This study is useful for accurately predicting cutting vibration and optimizing milling parameters, which is helpful for improving the process effects of micro-end-milling.

## 2. Experimental Conditions

**Figure 1:**
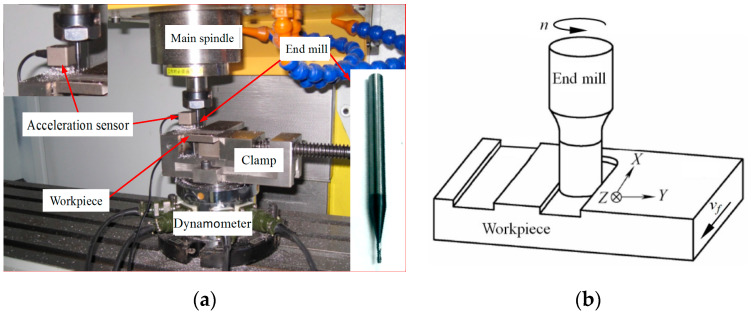
(**a**) Test setup. (**b**) coordinate system for micro-end-milling.

Figure 1 shows the test setup and coordinate system of micro-end-milling. The experiments of micro-end-milling of straight grooves were conducted on a SYIL S7 CNC milling machine (made by the SYIL CNC machine tools Co. Ltd., Ningbo, China). According to Figure 1a, the SDC-CJ4F dynamometer was mounted rigidly on the workbench of the milling machine, the clamp was attached to the top of the dynamometer, and the workpiece was fixed onto the clamp. The SDC-CJ4F dynamometer is a strain-type cutting force testing equipment with four channels. In the process of measurement, the signal of the cutting force was transformed into the current signal by the dynamometer, amplified by the FS21–4/6 amplifier and then entered into the computer by the INV3018A filter. A INV9832 three-component acceleration sensor was used to monitor the dynamic response of the micro-end-milling operation, where the sensitivity of the sensor was 10 mv/g, the maximum working acceleration was 500 g and the frequency range was 1–5 KHz. The acquired vibration signal was also entered into the computer through the INV3018A filter for further analysis, where the sampling frequency was 5000 Hz.

In order to have correct signal analysis, the coordinate system of micro-end-milling is shown in Figure 1b where *X*, *Y* and *Z* directions correspond, respectively to the feed direction, the width direction of the straight groove and the axis of the end mill; the coordinate origin is the intersection point of the axis of the end mill and the bottom machined surface.

As shown in Figure 1a, the cutting tool was a coated cemented carbide two-edged flat end mill. The percentages of WC and Co in the tool material were 90% and 10%, respectively. The grain size was 0.6 μm. The diameter of the end mill was 2 mm, its rake angle, flank angle and helical angle were 8°, 12° and 35°, respectively and the cutting-edge radius was 2 μm. The AISI 1045 steel (made by the Anyang iron & steel Co. Ltd., Anyang, China. Its chemical composition and properties are shown in Tables S1 and S2) was chosen as the workpiece material. The lubrication mode was dry cutting.

## 3. Waveform Characteristics of Cutting Vibration during Micro-End-Milling of Straight Groove

### 3.1. Characteristics of Time–Domain Response

**Figure 2:**
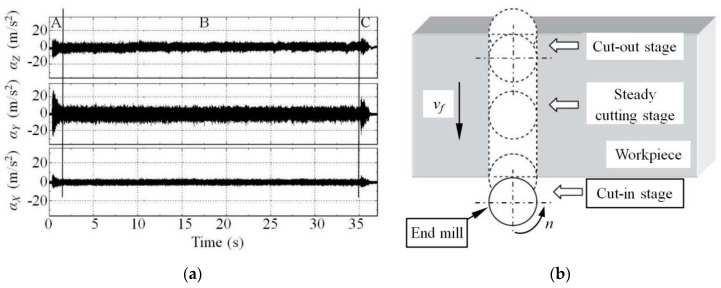
(**a**) Time–domain waveform of cutting vibration. (**b**) three stages of milling straight groove.

Figure 2 shows the time–domain waveforms of vibration measured during micro-end-milling of the straight groove, which represents the instantaneous values of acceleration as a function of time. The milling parameters in Figure 2 were as follows: the spindle speed *n* = 9000r/min, the feed velocity *v_f_* = 60 mm/min (namely the feed per tooth *f*_z_ = 3.33μm/z) and the axial depth of cut *a_p_* = 0.6 mm. the acceleration vibration along the *X*, *Y* and *Z* directions are the feed acceleration *α_X_*, the transverse acceleration *α_Y_* and the axial acceleration *α_Z_*, respectively.

According to Figure 2a, the whole time–domain waveform can be divided into three sections labeled as A, B and C, which correlate to the three stages of the milling process, namely the cut-in stage, steady cutting stage and cut-out stage as illustrated in Figure 2b. It should be noted that, during the cut-in and cut-out stages, the time–domain waveforms in sections A and C show clear transient characteristics with a larger peak value of acceleration vibration because of the dynamic interaction between the end mill and workpiece. During the steady cutting stage, the time–domain waveform in section B shows a uniform stabilized pattern with a lower peak value of acceleration.

From the time–domain waveform, the different characteristic variables of acceleration, such as the mean value, peak value and root mean square (RMS), etc., can be obtained, while the peak value is usually used as the analysis index for the cutting machining. According to Figure 2a, the peak value of accelerations along the *X*, *Y* and *Z* directions within the steady cutting stage are, respectively 12.08, 14.77 and 6.97 m/s^2^, which shows that, during micro-end-milling of the straight groove, the accelerations along the three directions from largest to smallest are in turn *α_Y_*, *α_X_* and *α_Z_*. The reason for this is that the axial stiffness of the end mill is far greater than the radial stiffness, which decreases the axial acceleration *α_Z_*, meanwhile the larger feed force along the *X* direction brings increased cutting damping which suppresses the increase of feed acceleration *α_X_*. Thus, the largest acceleration vibration usually appears in the *Y* direction, namely in the width direction of the straight groove.

### 3.2. Characteristics of Frequency Response

**Figure 3:**
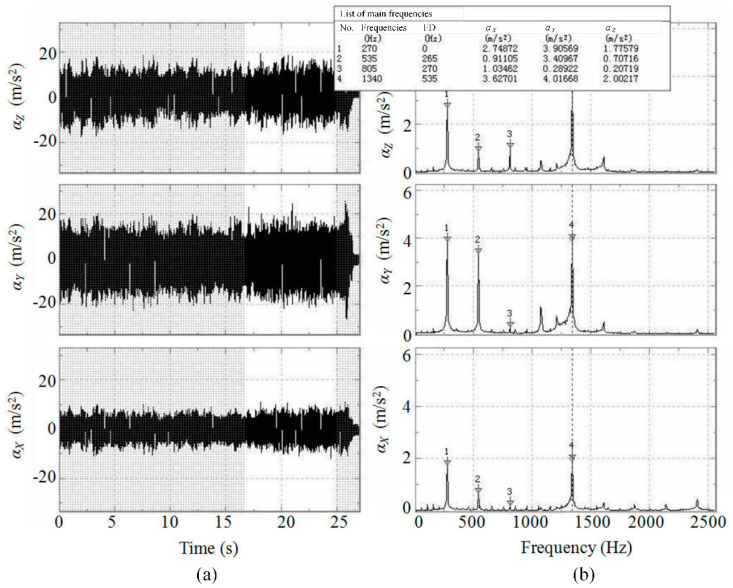
(**a**) Time–domain waveform of cutting vibration. (**b**) frequency response of cutting vibration.

Figure 3 shows the time–domain waveform and frequency response of the cutting vibration, where the frequency response is obtained by the Fourier transformation of the time–domain waveform. The signals correspond to a cutting condition of the spindle speed of 9000 r/min, the feed per tooth of 4.44 μm/z and axial depth of cut of 0.6 mm. The FD in Figure 3 represents the frequency differences between adjacent main frequencies.

According to Figure 3, with the given experimental parameters, the main frequencies of acceleration were measured to be 270, 535, 805 and 1340 Hz, with differences between frequencies being 265, 270 and 535 Hz, which are all close to 270 Hz or its frequency doubling. At the same test, the main frequencies of cutting force were 130.859, 261.719, 392.579 and 523.438, with frequency difference 130.859 (Seen from Figure S1). In theory, if the experiment for Figure 3 is in the situation of stable cutting, the main frequencies of both cutting vibration and cutting force should be equal to the spindle rotation frequency 150 Hz, cutting frequency 300Hz and their frequency doubling. However, due to the performance and installation of the sensor, deviations always occur between the theoretical and measured values and between the measured values of different sensors. However, it can be concluded by the comparison of frequency spectrums with different spindle speeds that the experimental machining processes are stable and do not appear to have significant cutting chatter. Thus, the process itself is the leading factor that influences the cutting vibration.

According to the above analysis, during micro-end-milling of the straight groove, the transverse acceleration *α_Y_* and the feed acceleration *α_X_* are all larger than the axial acceleration *α_Z_* and so these are the more important factors that should be considered for improving the machined quality and the tool life of micro-end-milling. Moreover, under the condition of steady milling, the milling process parameters can be considered to be the main factors that cause the increase of cutting vibration. Hence, the following research will focus mainly on the relationship of the milling parameters and accelerations *α_X_* and *α_Y_*.

## 4. Relationship of Milling Parameters and Acceleration Vibration

### 4.1. Influence of Milling Parameters on Acceleration Vibration

The single-factor experiment was used to analyze the relationship between milling parameters and acceleration vibration and Table 1 lists the chosen variables for the single-factor experiment. In Table 1, the underlined values indicate the chosen fixed values of milling parameters. In the process of conducting the experiments, two milling parameters were equal to their own fixed values and the influence rules of the third parameter on accelerations were then observed and analyzed.

**Figure 4:**
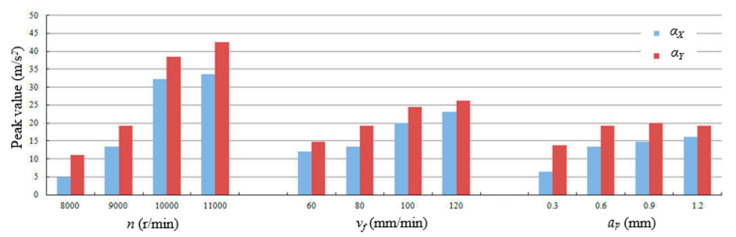
Influence of milling parameters on acceleration vibration *α_X_* and *α_Y_*.

Figure 4 shows the influence of milling parameters on the measured accelerations. As shown in Figure 4, under the experimental conditions, the transverse accelerations *α_Y_* were all greater than the feed acceleration *α_X_*, however *α_X_* and *α_Y_* have similar change trends. The influence of spindle speed *n* on accelerations *α_X_* and *α_Y_* was more significant than the feed velocity *v_f_* and axial depth of cut *a_p_*.

With the rise of spindle speed *n*, the accelerations *α_X_* and *α_Y_* gradually increase. The reason is mainly because if the feed velocity *v_f_* doesn’t change, the feed per tooth *f_z_* will constantly decrease with the increase of spindle speed *n* which causes the reduction of uncut chip thickness, so the “ploughing” action in the micro cutting area will be enhanced and the “cutting” action will be weakened, which brings the rapid increase of acceleration vibration.

With the rise of feed velocity *v_f_*, the accelerations *α_X_* and *α_Y_* continuously increase, the reason for this may be that the rise of feed velocity *v_f_* increases the feed per tooth *f_z_*, thus improving the dynamic cutting load. In addition, the rise of axial depth of cut *a_p_* will increase the damping in the width direction of the straight groove, thus the transverse acceleration *α_Y_* remains basically unchanged, even slightly reduced, when the axial depth of cut *a_p_* was more than 0.6 mm.

### 4.2. Correlation of Acceleration Vibration and Milling Parameters

In statistical analysis, the correlation coefficient is often used to quantitatively assess the relationship between two variables. The Spearman’s rank correlation coefficient *r_s_* is a nonparametric measure of statistical dependence between the ranking of two variables, and the value range of *r_s_* is from −1 to 1. If the |*r_s_*| is closer to 1, it indicates that there is a more significant correlation between two variables. The Spearman’s rank correlation coefficient *r_s_* between two variables *X* and *Y* can be computed from,

(1) $$r_{s}=\frac{\sum_{i=1}^{m}\left(x_{i}-\bar{x})(y_{i}-\bar{y}\right)}{\sqrt{\sum_{i=1}^{m}{\left(x_{i}-\bar{x}\right)}^{2}\sum_{i=1}^{m}{\left(y_{i}-\bar{y}\right)}^{2}}}$$

where *m* is the number of observations, *i* represents the positive integer from 1 to *m*, *x_i_* and *y_i_* are the *i*th value of rank variables *x* and *y* that are converted from raw variables *X* and *Y*, $\bar{x}$ and $\bar{y}$ are the mean values of rank variables *x* and *y* [31].

From the experimental results shown in Figure 4, the Spearman’s rank correlation between accelerations and milling parameters were computed and listed in Table 2. The correlation coefficient *r_s_* between *α_X_* and *α_Y_* is 0.993 that is almost close to 1 and the sig. (2-tailed) value is much less than the significant level of 0.01. This means that the *α_X_* and *α_Y_* are not independent of each other and there is actually a strong interdependence between them.

Further, the correlation coefficients between *α_X_*, *α_Y_* and *n* are all 0.767 and the sig. (2-tailed) values are 0.004, which means that there are statistically significant correlations between *α_X_*, *α_Y_* and *n*, and the change of spindle speed *n* strongly affects the accelerations *α_X_* and *α_Y_*. The correlation coefficients between *α_X_*, *α_Y_* and *v_f_* are 0.471 and the sig. (2-tailed) values are 0.122 which is more than the significant level of 0.01, so there are weak correlations between *α_X_*, *α_Y_* and *v_f_.* Similarly, the axial depth of cut *a_p_* has the weakest influence on accelerations *α_X_* and *α_Y_*.

## 5. Relationship Models between Milling Parameters and Acceleration Vibration

The experimental modeling approach is the most commonly used research approach in the field of mechanical manufacturing. Using limited experimental data, it can establish the relationship model between independent variables and dependent variables that can be used either to estimate quantitatively the relationships among variables or to predict new outcomes. In this section, the Taguchi experimental method and the regression analysis was used to establish the relationship models between milling parameters and the peak accelerations *α_X_* and *α_Y_*.

### 5.1. Taguchi Experiment and Its Result

The Taguchi method is a powerful technique of experimental design and it applies orthogonal arrays of statistically designed experiments to obtain the best results with a minimum number of experiments. In this experiment with three factors at four levels each, the fractional factorial design used was a standard L_16_(4^5^) orthogonal array [32,33]. Table 3 shows the L_16_ orthogonal matrix and the peak observed values of accelerations. Each row of the matrix represents one trial and the sequence in which these trials were carried out was randomized.

### 5.2. First Type of Relationship Model Based on Power Function

Micro-end-milling, like most of the cutting processes, is a kind of non-free oblique cutting technique. During non-free oblique cutting, the state parameters, such as cutting vibration, cutting force and cutting temperature, have evident nonlinear relationships with control parameters including cutting parameters, tool geometry angles, machined material, etc. In the applied research of metal cutting, the power function is often used to describe the relationship between state parameters and control parameters [32]. Hence, after the machine tool, cutting tool, workpiece material and lubrication mode were determined, the relationship model between acceleration *α* and milling parameters can be expressed as

(2) $$\alpha =k\cdot n^{x}\cdot v_{f}{}^{y}\cdot a_{p}{}^{z}$$

where *k*, *x*, *y* and *z* are a set of constant coefficients, and *k* depends mainly on the cutting tool, machined material and lubrication mode, etc.

Based on the observed values in Table 3, the constant coefficients *k*, *x*, *y* and *z* can be obtained by using the SPSS soft. The power function models between accelerations *α_X_*, *α_Y_* and milling parameters are found to be,

(3) $$\alpha_{X}=1.152\times 10^{-12}\cdot n^{3.167}\cdot v_{f}{}^{0.318}\cdot a_{p}{}^{0.262}$$

(4) $$\alpha_{Y}=4.607\times 10^{-10}\cdot n^{2.553}\cdot v_{f}{}^{0.308}\cdot a_{p}{}^{0.222}$$

From Equations (3) and (4), it can be observed that the three milling parameters have similar influence trends on the accelerations *α_X_* and *α_Y_* and that the influence of spindle speed *n* is more significant than the feed velocity *v_f_* and the axial depth of cut *H,* as also concluded from the single-factor experiments.

### 5.3. Second Type of Relationship Model Based on Second-Order Response Surface Model

The response surface methodology (RSM) is a collection of mathematical and statistical techniques for building empirical models. Because the second-order response surface model is known to have high flexibility and can take on a wide variety of functional forms, it is often used as an approximation to the true response surface [32,34]. In general, the second-order response surface equation is given by,

(5) $$y=\beta_{0}+\sum_{i=1}^{n}\beta_{i}x_{i}+\sum_{i=1}^{n}\beta_{ii}x_{i}{}^{2}+\sum_{i<j}\sum_{j=2}^{n}\beta_{ij}x_{i}x_{j}$$

where *y* is the dependent variable, *x_i_* or *x_j_* are the independent variables and the *β*’s are a set of unknown constant coefficients. Based on Equation (5), the second-order response surface model between the acceleration vibration *α* and milling parameters can be expressed as,

(6) $$\alpha =k_{0}+k_{1}n+k_{2}v_{f}+k_{3}a_{p}+k_{4}n^{2}+k_{5}v_{f}{}^{2}+k_{6}a_{p}{}^{2}+k_{7}nv_{f}+k_{8}na_{p}+k_{9}v_{f}a_{p}$$

where *k*_0_-*k*_9_ are a set of constant coefficients that can be solved by the nonlinear regression analysis. The resulting response surface models of *α_X_* and *α_Y_* were found to be,

(7) $$\begin{array}{ll} \alpha_{X}= & -304.761+0.063n+0.322v_{f}-61.072a_{p}-3.273\times 10^{-6}n^{2}\\ & -2.847a_{p}{}^{2}-1.477\times 10^{-5}nv_{f}+0.009na_{p}-0.127v_{f}a_{p} \end{array}$$

(8) $$\begin{array}{ll} \alpha_{Y}= & -239.964+0.045n+0.34v_{f}+13.071a_{p}-1.806\times 10^{-6}n^{2}+0.005v_{f}{}^{2}\\ & -10.722a_{p}{}^{2}-8.964\times 10^{-5}nv_{f}+0.003na_{p}-0.296v_{f}a_{p} \end{array}$$

### 5.4. Comparison of the Two Types of Relationship Models

Table 4, Table 5, Table 6 and Table 7 are the analysis of variance (ANOVA) of the above four relationship models, where “SS”, “DOF” and “MS” represent separately the sum of square, degree of freedom and mean square and the “R^2^” function is known as the coefficient of determination, which has a range from 0 to 1. The larger the coefficient of determination, the better the goodness-of-fit of the model. From Table 4, Table 5, Table 6 and Table 7, the “R^2^” of the power function models for *α_X_* and *α_Y_* are separately 0.607 and 0.708, while the “R^2^” of the response surface models for *α_X_* and *α_Y_* are 0.825 and 0.881. The results of ANOVA show that the response surface models are more sensitive and have a better fitting effect than the power function models and the models of *α_Y_* have better goodness-of-fit than the models of *α_X_*.

**Figure 5:**
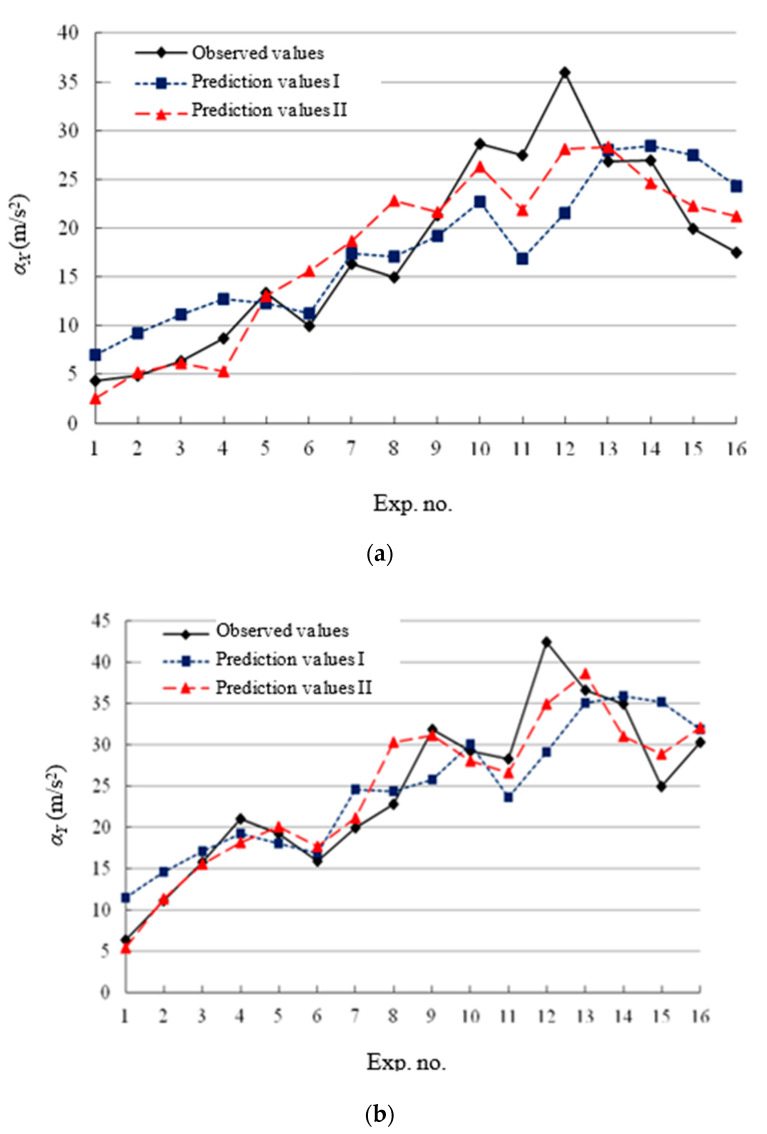
Comparison between prediction values and observed values. (**a**) feed acceleration vibration *α_X_*. (**b**) transverse acceleration vibration *α_Y_*.

Figure 5 shows the comparison between prediction values and observed values, where the horizontal axis represents the same experiment number with Table 3, and the prediction values Ⅰ and Ⅱ are, respectively the prediction results from the power function models and response surface models. According to Figure 5, The deviation between prediction values Ⅱ and observed values are significantly less than that of the prediction values Ⅰ, so the response surface model has higher prediction accuracy than the power function model, which makes it more suitable for the control and prediction of cutting vibration during micro-end-milling.

## 6. Conclusions

During micro-end-milling of the straight groove, the components of acceleration vibration from largest to smallest are in turn the transverse acceleration *α_Y_*, the feed acceleration *α_X_* and the axial acceleration *α_Z_*. Hence, the transverse acceleration vibration *α_Y_* should be controlled and suppressed first, which is helpful for improving the accuracy of the groove width and machined surface roughness.

Under the conditions of steady micro-end-milling, the change of spindle speed *n* strongly affects the acceleration vibration, however there are weak correlations between acceleration vibration and feed velocity *v_f_*, axial depth of cut *a_p_*. Therefore, during micro end-milling, the spindle speed is the most important parameter that should be controlled, and the reduction of spindle speed can evidently decrease cutting vibration.

Based on the power function and second-order response surface equation, two types of relationship models between acceleration vibration and milling parameters were established. Compared with the power function model, the response surface model has higher prediction accuracy, thus it is more suitable for the prediction of cutting vibration and the optimization of milling parameters during micro-end-milling of the straight groove.

The analytical modeling of acceleration vibration developed in this research can be used not only for milling straight grooves, but also for milling side face, step-faceting, etc. and further, it can be applied in micro ball end-milling.

## Figures and Tables

**Table 1:** Milling parameters of the single-factor experiment.

Milling Parameter	Value
Spindle speed *n* (r/min)	8000, 9000, 10000, 11,000
Feed velocity *v_f_* (mm/min)	60, 80, 100, 120 (Feed per tooth *f*_z_ is in between 2.727 and 7.5 μm/z)
Axial depth of cut *a_p_* (mm)	0.3, 0.6, 0.9, 1.2

**Table 2:** Correlation between acceleration vibration and milling parameters.

		*α_X_*	*α_Y_*	*n*	*v_f_*	*a_p_*
*α_X_*	Correlation coefficient Sig. (2-tailed)	1.000 –	0.993 ^**^ **0.000**	0.767 ^**^ **0.004**	**0.471** **0.122**	**0.314** **0.320**
*α_Y_*	Correlation coefficient Sig. (2-tailed)	0.993 ^**^ 0.000	1.000 –	0.767 ^**^ **0.004**	**0.471** **0.122**	**0.305** **0.335**
*n*	Correlation coefficient Sig. (2-tailed)	0.767 ^**^ 0.004	0.767 ^**^ 0.004	1.000 –	−0.036 0.911	−0.036 0.911
*v_f_*	Correlation coefficient Sig. (2-tailed)	0.471 0.122	0.471 0.122	−0.036 0.911	1.000 –	−0.036 0.911
*a_p_*	Correlation coefficient Sig. (2-tailed)	0.314 0.320	0.305 0.335	−0.036 0.911	−0.036 0.911	1.000 –

** Correlation is significant at the 0.01 level.

**Table 3:** L_16_ orthogonal matrix and peak observed values of accelerations.

Experimental No.	Factors and Levels			Observed Values (Peak)	
	*n* (r/min)	*v_f_* (mm/min)	*a_p_* (mm)	*α_X_* (m/s^2^)	*α_Y_* (m/s^2^)
1	8000	60	0.3	4.3743	6.2874
2	8000	80	0.6	4.9289	11.0619
3	8000	100	0.9	6.3900	15.7958
4	8000	120	1.2	8.6652	21.0226
5	9000	60	0.6	13.4141	19.2034
6	9000	80	0.3	9.9442	15.9355
7	9000	100	1.2	16.3226	19.9135
8	9000	120	0.9	14.9139	22.8469
9	10,000	60	0.9	21.3497	31.8995
10	10,000	80	1.2	28.6551	29.2786
11	10,000	100	0.3	27.4740	28.3054
12	10,000	120	0.6	35.9891	42.5184
13	11,000	60	1.2	26.8941	36.6257
14	11,000	80	0.9	26.9787	34.9297
15	11,000	100	0.6	19.9663	24.9208
16	11,000	120	0.3	17.5018	30.3683

**Table 4:** ANOVA for the power function model of *α_X_*.

Source	SS	DOF	MS
Regression	5868.953	4	1467.238
Residual	541.139	12	45.095
Uncorrected total	6410.092	16	–
Corrected total	1377.540	15	–
a. R^2^ = 1 − (Residual SS)/(Corrected SS) = 0.607			

**Table 5:** ANOVA for the power function model of *α_Y_*.

Source	SS	DOF	MS
Regression	10,564.451	4	2641.113
Residual	418.772	12	34.898
Uncorrected total	10,983.223	16	–
Corrected total	1432.394	15	–
a. R^2^ = 1 − (Residual SS)/(Corrected SS) = 0.708			

**Table 6:** ANOVA for the response surface model of *α_X_*.

Source	SS	DOF	MS
Regression	6169.337	10	616.934
Residual	240.756	6	40.126
Uncorrected total	6410.092	16	–
Corrected total	1377.540	15	–
a. R^2^ = 1 − (Residual SS)/(Corrected SS) = 0.825			

**Table 7:** ANOVA for the response surface model of α*_Y_*.

Source	SS	DOF	MS
Regression	10,813.006	10	1081.301
Residual	170.217	6	28.369
Uncorrected total	10,983.223	16	–
Corrected total	1432.394	15	–
a. R^2^ =1 − (Residual SS)/(Corrected SS) = 0.881			

## Supplementary Materials

The following are available online at https://www.mdpi.com/2072-666X/11/5/494/s1, Figure S1: The amplitude spectrum of feed force *F*_f_ with different spindle speed when the feed per tooth is 4.44 μm/z and axial depth of cut is 0.6 mm. Table S1: Chemical composition of AISI 1045 steel. Table S2: Properties of AISI 1045 steel.
