# Supplementary material for: Experimental Investigation of Cutting Vibration during Micro-End-Milling of the Straight Groove
Source: Micromachines (Basel). 2020 May 13;11(5):494. doi: 10.3390/mi11050494 (PMC7281379; doi:10.3390/mi11050494)
Supplement: Supplementary file 1 [file micromachines-11-00494-s001.pdf]

micromachines

Supplementary Materials

Table S1. Chemical composition of AISI 1045 steel.

Elements	wt%	Elements	wt%	Elements	wt%
C	0.42–0.50	Ni	≤0.25	Si	0.17–0.37
Cr	≤0.25	P	≤0.035	Cu	≤0.25
Mn	0.50–0.80	S	≤0.035	Fe	Other

Table S2. Properties of AISI 1045 steel.

Tensile strength σ_b	≥600 Mpa	Brinell hardness HB	≤197
yield strength σ_s	≥355 Mpa	Modulus of elasticity E	210 Gpa
Extensibility δ	≥16%	Poisson ratio ν	0.269
Reduction of crosssection area ψ	≥40%	Density ϱ	7.85 g/cm^3

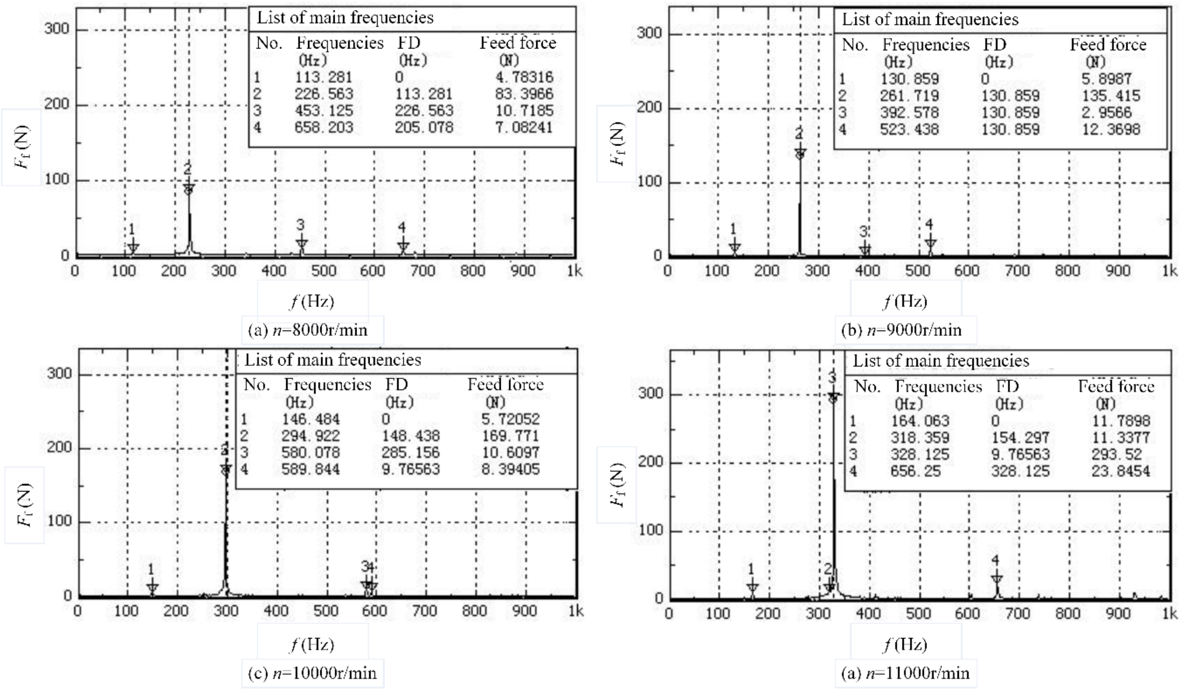

Figure S1. The amplitude spectrum of feed force F_f with different spindle speed when the feed per tooth is 4.44 μm/z and axial depth of cut is 0.6 mm.
